# Non‐convulsive epilepsy with acute‐onset and short‐lasting repeated fatigue attacks: A case of 30‐year‐old man

**DOI:** 10.1002/jgf2.531

**Published:** 2022-02-28

**Authors:** Takashi Akimoto, Tadashi Kobayashi, Hiroki Maita, Hiroshi Osawa, Hiroyuki Kato

**Affiliations:** ^1^ 92149 Department of General Medicine Hirosaki University School of Medicine and Hospital Hirosaki Japan; ^2^ Development of Community Healthcare Hirosaki University Graduate School of Medicine Hirosaki Japan; ^3^ General Medicine Hirosaki University Graduate School of Medicine Hirosaki Japan

**Keywords:** acute‐onset and short‐lasting, focal epilepsy, general malaise, non‐convulsive seizure, partial seizure, recurrent fatigue

## Abstract

A 30‐year‐old man was referred to our department because of repeated acute‐onset and short‐lasting fatigue attacks, which occurred from 3 months before the referral. He had no abnormal findings in blood tests, electrocardiogram (including 24‐ h monitoring), or head MRI (including angiography). His vital signs were unremarkable, and his physical examination revealed no abnormal findings. Detailed history‐taking with closed‐ended questions revealed the occurrence of tingling sensation from the right fingers as the aura before his attacks. Electroencephalography was performed, which revealed focal epilepsy. Levetiracetam resolved his symptoms. Physicians could consider non‐convulsive epilepsy as a potential cause of repeated acute‐onset and short‐lasting fatigue attacks of unknown etiology after underlying conditions, such as metabolic diseases, have been ruled out.

## 1 INTRODUCTION

Fatigue is the sixth most common subjective symptom in Japan.^1^ In the primary care setting, patients often present with fatigue as the chief complaint.^2^ However, there are few cases of recurrent acute‐onset fatigue episodes that improve quickly. Epilepsy is a common disease with an annual prevalence of 4–7 per 10,000 individuals in developed countries.^3^ It is often overlooked in patients without characteristic symptoms, such as loss of consciousness or generalized convulsions.^4^ Here, we report a case of non‐convulsive idiopathic epilepsy diagnosed by detailed history‐taking, including closed‐ended questions, in which the aura was a tingling sensation in the right hand.

## 2 CASE

A 30‐year‐old man with no history of illness visited his previous physician because of fatigue for 3 months prior to his visit to our department. His blood tests, ECG (including Holter monitoring), head MRI, and magnetic resonance angiography findings revealed no abnormalities. The previous physician suspected cervical dizziness and syncope. However, the patient aimed to investigate the cause further and was referred to our hospital. On his first visit to our department, his blood pressure was 129/55 mmHg, his pulse rate was 70 beats/min, and his other vital signs were unremarkable. On physical examination, there were no findings of muscle weakness, dysesthesia, or skin rash. Blood test results revealed no abnormalities that could have caused fatigue (Table 1). He had no regular oral medication and no family history of epilepsy. During the medical interview, his history revealed an acute‐onset fatigue that lasted for approximately 1 min with the same intensity and gradually improved over approximately 5 min regardless of the time or place. The symptoms occurred approximately once a week, and there was no time of day or exacerbating factor that made it more likely to occur. The patient remained conscious when these symptoms appeared. When we asked him “Are there any symptoms that bothered you before the onset of fatigue?,” he responded that he had experienced a tingling sensation from the right index finger to the little finger before the acute‐onset fatigue. Therefore, together with the history of acute intermittent fatigue attacks, we suspected focal epilepsy and performed an EEG examination that showed abnormal sharp waves (isolated short burst (4–5 Hz), localized in the anterior and mid‐temporal areas) (Figure S1). We diagnosed the patient with non‐convulsive idiopathic epilepsy and treated him with 500 mg/day levetiracetam. Subsequently, the dose increased to 1000 mg/day. The acute‐onset fatigue disappeared, and the EEG performed 2 years later was normalized. The patient is currently under follow‐up examination.

**TABLE 1:** Blood test results of the case patient

	Patient's data	Reference range
White blood cell count (10^3^/μL)	5.60	3.30–8.60
Red blood cell count (10^6^/μL)	5.44	4.35–5.55
Hemoglobin concentration (g/dL)	**16.9**	13.7–16.8
Mean corpuscular volume (fL)	86.4	83.6–98.2
Serum sodium concentration (mmol/L)	142	138–145
Serum potassium concentration (mmol/L)	3.9	3.6–4.8
Serum iron concentration (μg/dL)	82	40–188
Serum ferritin concentration (ng/mL)	188	50–200
Thyroid‐stimulating hormone (μIU/mL)	1.02	0.50–5.00
Free triiodothyronine (pg/mL)	3.51	2.30–4.00
Free thyroxine (ng/mL)	1.70	0.90–1.70

Note

Bold number indicates a value outside the reference range.

## 3 DISCUSSION

We found that repeated episodes of acute‐onset and short‐lasting fatigue could be attributed to non‐convulsive epilepsy; hence, it is important to confirm the signs using a closed‐ended question.

Medical history is crucial for distinguishing epilepsy from non‐epileptic manifestations,^5^ and confirming non‐convulsive epilepsy is more difficult than confirming convulsive epilepsy.^4^ We suspected a possibility of non‐convulsive epilepsy in this case owing to detailed history‐taking, wherein the patient described the occurrence of acute fatigue regardless of location, short and transient (approximately 1 min long) symptoms, and appearance of abnormal sensation in the right hand before fatigue onset.

Acute‐onset and quickly resolving symptoms could be caused by transient ischemic attacks (TIA), migraine, syncope, cataplexy, paroxysmal dystonia, or epilepsy.^5^ In all such cases, history‐taking is important: TIA is characterized by transient neurological abnormalities,^6^ migraine is accompanied by symptoms lasting 4–72 h,^7^ transient loss of consciousness is observed in syncope,^8^ cataplexy is characterized by episodes of muscle weakness that occur during wakefulness and are triggered by emotions,^9^ and paroxysmal dystonia is triggered by muscle movement or physical activity.^10^ In our case, there were no neurological abnormalities, loss of consciousness, and emotions or physical activity that can be triggered, and the symptoms lasted only several minutes.

History‐taking with open‐ended and closed‐ended questions confirms the aura of generalized epilepsy in 21.3% and 64.3% of patients, respectively.^11^ If epilepsy is suspected, the closed‐ended questions should be used preferably. In our case, we asked the patient if there was any aura before the fatigue in the medical interview using a closed‐ended question and found the incidence of the abnormal sensation in the right hand. Appropriate history‐taking requires a great deal of knowledge regarding diseases,^5^ and physicians should engage in continuous self‐learning.

Repeated fatigue attacks with acute‐onset and short‐lasting symptoms may be caused by non‐convulsive epilepsy. Detailed history‐taking with closed‐ended questions to confirm the aura may be useful in confirming non‐convulsive epilepsy. Physicians should consider non‐convulsive epilepsy as a possible cause of repeated fatigue attacks of unknown etiology.

## CONFLICT OF INTERESTS

The authors declare no conflict of interests for this article.

## INFORMED CONSENT

Written informed consent was obtained from the patient for the publication of this case report.

## Supporting information

Fig S1
