# Supplementary figures and images for: Non‐convulsive epilepsy with acute‐onset and short‐lasting repeated fatigue attacks: A case of 30‐year‐old man
Source: J Gen Fam Med. 2022 Feb 28;23(4):275–7. doi: 10.1002/jgf2.531 (PMC9249925; doi:10.1002/jgf2.531)

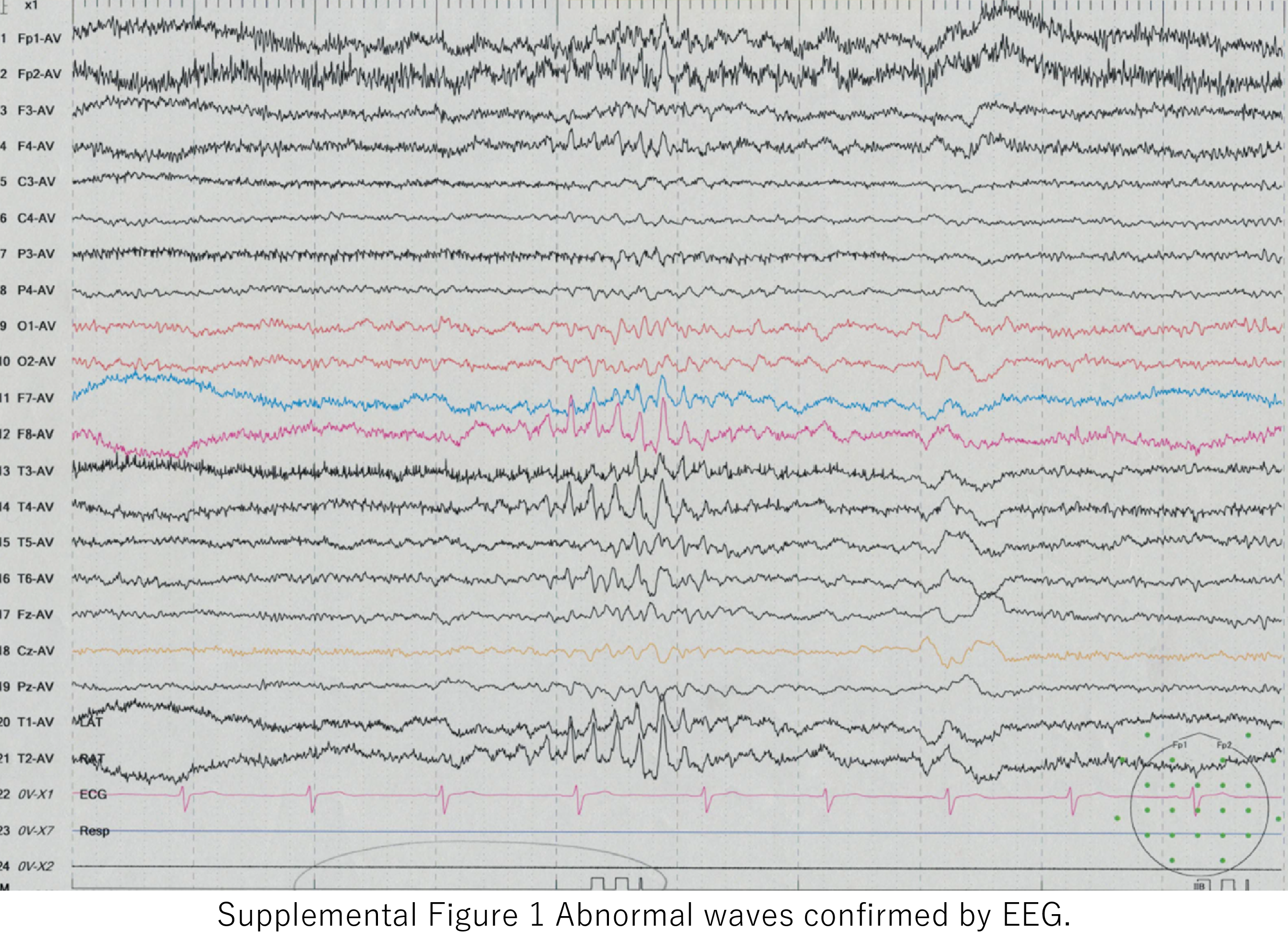

Supplement: Supplementary file 1 — Fig S1 [file JGF2-23-275-s001.tiff]
